# Supplementary material for: A systematic review on the detection of volatile organic compounds in exhaled breath in experimental animals in the context of gastrointestinal and hepatic diseases
Source: PLoS One. 2023 Sep 21;18(9):e0291636. doi: 10.1371/journal.pone.0291636 (PMC10513283; doi:10.1371/journal.pone.0291636)
Supplement: S1 File — (DOCX) [file pone.0291636.s002.docx]

***Table 4: Search stategy***

***Pubmed***

(“animal experimentation”[MeSH Terms] OR “models, animal”[MeSH Terms] OR “invertebrates”[MeSH Terms] OR “Animals”[Mesh:noexp] OR “animal population groups”[MeSH Terms] OR “chordata”[MeSH Terms:noexp] OR “chordata, nonvertebrate”[MeSH Terms] OR “vertebrates”[MeSH Terms:noexp] OR “amphibians”[MeSH Terms] OR “birds”[MeSH Terms] OR “fishes”[MeSH Terms] OR “reptiles”[MeSH Terms] OR “mammals”[MeSH Terms:noexp] OR “primates”[MeSH Terms:noexp] OR “artiodactyla”[MeSH Terms] OR “carnivora”[MeSH Terms] OR “cetacea”[MeSH Terms] OR “chiroptera”[MeSH Terms] OR “elephants”[MeSH Terms] OR “hyraxes”[MeSH Terms] OR “insectivora”[MeSH Terms] OR “lagomorpha”[MeSH Terms] OR “marsupialia”[MeSH Terms] OR “monotremata”[MeSH Terms] OR “perissodactyla”[MeSH Terms] OR “rodentia”[MeSH Terms] OR “scandentia”[MeSH Terms] OR “sirenia”[MeSH Terms] OR “xenarthra”[MeSH Terms] OR “haplorhini”[MeSH Terms:noexp] OR “strepsirhini”[MeSH Terms] OR “platyrrhini”[MeSH Terms] OR “tarsii”[MeSH Terms] OR “catarrhini”[MeSH Terms:noexp] OR “cercopithecidae”[MeSH Terms] OR “hylobatidae”[MeSH Terms] OR “hominidae”[MeSH Terms:noexp] OR “gorilla gorilla”[MeSH Terms] OR “pan paniscus”[MeSH Terms] OR “pan troglodytes”[MeSH Terms] OR “pongo pygmaeus”[MeSH Terms]) OR ((animals[tiab] OR animal[tiab] OR mice[Tiab] OR mus[Tiab] OR mouse[Tiab] OR murine[Tiab] OR woodmouse[tiab] OR rats[Tiab] OR rat[Tiab] OR murinae[Tiab] OR muridae[Tiab] OR cottonrat[tiab] OR cottonrats[tiab] OR hamster[tiab] OR hamsters[tiab] OR cricetinae[tiab] OR rodentia[Tiab] OR rodent[Tiab] OR rodents[Tiab] OR pigs[Tiab] OR pig[Tiab] OR swine[tiab] OR swines[tiab] OR piglets[tiab] OR piglet[tiab] OR boar[tiab] OR boars[tiab] OR “sus scrofa”[tiab] OR ferrets[tiab] OR ferret[tiab] OR polecat[tiab] OR polecats[tiab] OR “mustela putorius”[tiab] OR “guinea pigs”[Tiab] OR “guinea pig”[Tiab] OR cavia[Tiab] OR callithrix[Tiab] OR marmoset[Tiab] OR marmosets[Tiab] OR cebuella[Tiab] OR hapale[Tiab] OR octodon[Tiab] OR chinchilla[Tiab] OR chinchillas[Tiab] OR gerbillinae[Tiab] OR gerbil[Tiab] OR gerbils[Tiab] OR jird[Tiab] OR jirds[Tiab] OR merione[Tiab] OR meriones[Tiab] OR rabbits[Tiab] OR rabbit[Tiab] OR hares[Tiab] OR hare[Tiab] OR diptera[Tiab] OR flies[Tiab] OR fly[Tiab] OR dipteral[Tiab] OR drosophila[Tiab] OR drosophilidae[Tiab] OR cats[Tiab] OR cat[Tiab] OR carus[Tiab] OR felis[Tiab] OR nematoda[Tiab] OR nematode[Tiab] OR nematodes[Tiab] OR sipunculida[Tiab] OR dogs[Tiab] OR dog[Tiab] OR canine[Tiab] OR canines[Tiab] OR canis[Tiab] OR sheep[Tiab] OR sheeps[Tiab] OR mouflon[Tiab] OR mouflons[Tiab] OR ovis[Tiab] OR goats[Tiab] OR goat[Tiab] OR capra[Tiab] OR capras[Tiab] OR rupicapra[Tiab] OR rupicapras[Tiab] OR chamois[Tiab] OR haplorhini[Tiab] OR monkey[Tiab] OR monkeys[Tiab] OR anthropoidea[Tiab] OR anthropoids[Tiab] OR saguinus[Tiab] OR tamarin[Tiab] OR tamarins[Tiab] OR leontopithecus[Tiab] OR hominidae[Tiab] OR ape[Tiab] OR apes[Tiab] OR “pan paniscus”[Tiab] OR bonobo[Tiab] OR bonobos[Tiab] OR “pan troglodytes”[Tiab] OR gibbon[Tiab] OR gibbons[Tiab] OR siamang[Tiab] OR siamangs[Tiab] OR nomascus[Tiab] OR symphalangus[Tiab] OR chimpanzee[Tiab] OR chimpanzees[Tiab] OR prosimian[Tiab] OR prosimians[Tiab] OR “bush baby”[Tiab] OR bush babies[Tiab] OR galagos[Tiab] OR galago[Tiab] OR pongidae[Tiab] OR gorilla[Tiab] OR gorillas[Tiab] OR “pongo pygmaeus”[Tiab] OR orangutan[Tiab] OR orangutans[Tiab] OR lemur[Tiab] OR lemurs[Tiab] OR lemuridae[Tiab] OR horse[Tiab] OR horses[Tiab] OR equus[Tiab] OR cow[Tiab] OR calf[Tiab] OR bull[Tiab] OR chicken[Tiab] OR chickens[Tiab] OR gallus[Tiab] OR quail[Tiab] OR bird[Tiab] OR birds[Tiab] OR quails[Tiab] OR poultry[Tiab] OR poultries[Tiab] OR fowl[Tiab] OR fowls[Tiab] OR reptile[Tiab] OR reptilia[Tiab] OR reptiles[Tiab] OR snakes[Tiab] OR snake[Tiab] OR lizard[Tiab] OR lizards[Tiab] OR alligator[Tiab] OR alligators[Tiab] OR crocodile[Tiab] OR crocodiles[Tiab] OR turtle[Tiab] OR turtles[Tiab] OR amphibian[Tiab] OR amphibians[Tiab] OR amphibia[Tiab] OR frog[Tiab] OR frogs[Tiab] OR bombina[Tiab] OR salientia[Tiab] OR toad[Tiab] OR toads[Tiab] OR “epidalea calamita”[Tiab] OR salamander[Tiab] OR salamanders[Tiab] OR eel[Tiab] OR eels[Tiab] OR fish[Tiab] OR fishes[Tiab] OR pisces[Tiab] OR catfish[Tiab] OR catfishes[Tiab] OR siluriformes[Tiab] OR arius[Tiab] OR heteropneustes[Tiab] OR sheatfish[Tiab] OR perch[Tiab] OR perches[Tiab] OR percidae[Tiab] OR perca[Tiab] OR trout[Tiab] OR trouts[Tiab] OR char[Tiab] OR chars[Tiab] OR salvelinus[Tiab] OR minnow[Tiab] OR cyprinidae[Tiab] OR carps[Tiab] OR carp[Tiab] OR zebrafish[Tiab] OR zebrafishes[Tiab] OR goldfish[Tiab] OR goldfishes[Tiab] OR guppy[Tiab] OR guppies[Tiab] OR chub[Tiab] OR chubs[Tiab] OR tinca[Tiab] OR barbels[Tiab] OR barbus[Tiab] OR pimephales[Tiab] OR promelas[Tiab] OR “poecilia reticulata”[Tiab] OR mullet[Tiab] OR mullets[Tiab] OR eel[Tiab] OR eels[Tiab] OR seahorse[Tiab] OR seahorses[Tiab] OR mugil curema[Tiab] OR atlantic cod[Tiab] OR shark[Tiab] OR sharks[Tiab] OR catshark[Tiab] OR anguilla[Tiab] OR salmonid[Tiab] OR salmonids[Tiab] OR whitefish[Tiab] OR whitefishes[Tiab] OR salmon[Tiab] OR salmons[Tiab] OR sole[Tiab] OR solea[Tiab] OR lamprey[Tiab] OR lampreys[Tiab] OR pumpkinseed[Tiab] OR sunfish[Tiab] OR sunfishes[Tiab] OR tilapia[Tiab] OR tilapias[Tiab] OR turbot[Tiab] OR turbots[Tiab] OR flatfish[Tiab] OR flatfishes[Tiab] OR sciuridae[Tiab] OR squirrel[Tiab] OR squirrels[Tiab] OR chipmunk[Tiab] OR chipmunks[Tiab] OR suslik[Tiab] OR susliks[Tiab] OR vole[Tiab] OR voles[Tiab] OR lemming[Tiab] OR lemmings[Tiab] OR muskrat[Tiab] OR muskrats[Tiab] OR lemmus[Tiab] OR otter[Tiab] OR otters[Tiab] OR marten[Tiab] OR martens[Tiab] OR martes[Tiab] OR weasel[Tiab] OR badger[Tiab] OR badgers[Tiab] OR ermine[Tiab] OR mink[Tiab] OR minks[Tiab] OR sable[Tiab] OR sables[Tiab] OR gulo[Tiab] OR gulos[Tiab] OR wolverine[Tiab] OR wolverines[Tiab] OR mustela[Tiab] OR llama[Tiab] OR llamas[Tiab] OR alpaca[Tiab] OR alpacas[Tiab] OR camelid[Tiab] OR camelids[Tiab] OR guanaco[Tiab] OR guanacos[Tiab] OR chiroptera[Tiab] OR chiropteras[Tiab] OR bat[Tiab] OR bats[Tiab] OR fox[Tiab] OR foxes[Tiab] OR iguana[Tiab] OR iguanas[Tiab] OR xenopus laevis[Tiab] OR parakeet[Tiab] OR parakeets[Tiab] OR parrot[Tiab] OR parrots[Tiab] OR donkey[Tiab] OR donkeys[Tiab] OR mule[Tiab] OR mules[Tiab] OR zebra[Tiab] OR zebras[Tiab] OR shrew[Tiab] OR shrews[Tiab] OR bison[Tiab] OR bisons[Tiab] OR buffalo[Tiab] OR buffaloes[Tiab] OR deer[Tiab] OR deers[Tiab] OR bear[Tiab] OR bears[Tiab] OR panda[Tiab] OR pandas[Tiab] OR “wild hog”[Tiab] OR “wild boar”[Tiab] OR fitchew[Tiab] OR fitch[Tiab] OR beaver[Tiab] OR beavers[Tiab] OR jerboa[Tiab] OR jerboas[Tiab] OR capybara[Tiab] OR capybaras[Tiab] OR canine [tiab] OR bovine [tiab] OR porcine [tiab] OR hog [tiab] OR hogs [tiab]) NOT medline[sb]) AND ("Breath Tests"[MeSH Terms] OR "Volatile Organic Compounds"[MeSH Terms] OR "Electronic Nose"[MeSH Terms] OR volatile organic compound*[Tiab] OR VOC[Tiab] OR VOCs[Tiab] OR volatile compound*[Tiab] OR volatile marker*[Tiab] OR volatile biomarker*[Tiab] OR biomarker indicator compound*[Tiab] OR electronic nose*[Tiab] OR e nose*[Tiab] OR eNose*[Tiab] OR nano artifical nose[Tiab] OR NA nose[Tiab] OR exhaled air[Tiab] OR breath*[Tiab] OR gas analys*[Tiab] OR gas metabolite*[Tiab] OR electronic aroma detection[Tiab] OR metabolomic*[Tiab] OR colorimetric sensor array*[Tiab] OR sensor array*[Tiab] OR conducting polymer*[Tiab] OR conducting polymere*[Tiab] OR spironose[Tiab] OR artificial olfaction[Tiab] OR metal oxide semiconductor*[Tiab] OR quartz microbalance*[Tiab] OR surface acoustic wave*[Tiab] OR biosensor*[Tiab]) AND ("digestive system"[MeSH Terms] OR "digestive system diseases"[MeSH Terms] OR "signs and symptoms, digestive"[MeSH Terms] OR "Sepsis"[Mesh] OR digestive system[Tiab] OR digestive disease*[Tiab] OR gastrointestinal[Tiab] OR biliary atresia[Tiab] OR biliary fistura[Tiab] OR cholangitis[Tiab] OR choledochal cyst*[Tiab] OR caroli disease[Tiab] OR cholestas*[Tiab] OR mirizzi[Tiab] OR alagille syndrome*[Tiab] OR biliary dyskinesia[Tiab] OR sphincter of oddi dysfunction[Tiab] OR choledocholithias*[Tiab] OR pancreaticobiliary maljunction*[Tiab] OR anorectal malformation[Tiab] OR imperforate anus[Tiab] OR diaphragmatic eventration*[Tiab] OR esophageal atresia*[Tiab] OR intestinal atresia*[Tiab] OR duodenal atresia*[Tiab] OR median arcuate ligament syndrome*[Tiab] OR esophageal fistula*[Tiab] OR tracheoesophageal fistula*[Tiab] OR gastric fistula*[Tiab] OR intestinal fistula*[Tiab] OR rectal fistula*[Tiab] OR anal fistula*[Tiab] OR perianal fistula*[Tiab] OR rectovaginal fistula*[Tiab] OR gastrointestinal neoplasm*[Tiab] OR gastrointestinal cancer*[Tiab] OR gastrointestinal carcinoma*[Tiab] OR gastrointestinal stromal tum*[Tiab] OR GIST*[Tiab] OR digestive system neoplasm*[Tiab] OR intestinal neoplasm*[Tiab] OR intestinal cancer*[Tiab] OR intestinal carcinoma*[Tiab] OR bile duct neoplasm*[Tiab] OR bile duct cancer*[Tiab] OR bile duct carcinoma*[Tiab] OR biliary tract neoplasm*[Tiab] OR biliary tract cancer*[Tiab] OR biliary tract carcinoma*[Tiab] OR cholangiocellular carcinoma*[Tiab] OR cholangiocarcinoma*[Tiab] OR gallbladder neoplasm*[Tiab] OR gallbladder cancer*[Tiab] OR gallbladder carcinoma*[Tiab] OR gallbladder adenocarcinoma*[Tiab] OR esophageal neoplasm*[Tiab] OR esophageal cancer*[Tiab] OR esophageal carcinoma*[Tiab] OR esophageal squamous cell carcinoma*[Tiab] OR esophageal adenocarcinoma*[Tiab] OR gastric neoplasm*[Tiab] OR gastric cancer*[Tiab] OR carcinoma ventriculi[Tiab] OR gastric carcinoma*[Tiab] OR gastric adenocarcinoma*[Tiab] OR gastric squamous cell carcinoma*[Tiab] OR stomach neoplasm*[Tiab] OR stomach cancer*[Tiab] OR stomach carcinoma*[Tiab] OR duodenal cancer*[Tiab] OR duodenal carcinoma*[Tiab] OR duodenal adenocarcinoma*[Tiab] OR ileal neoplasm*[Tiab] OR ileum carcinoma*[Tiab] OR jejunal neoplasm*[Tiab] OR jejunal carcinoma[Tiab] OR jejunum neoplasm*[Tiab] OR jejunum carcinoma[Tiab] OR cecal neoplasm*[Tiab] OR cecal cancer*[Tiab] OR cecal carcinoma*[Tiab] OR cecal adenocarcinoma*[Tiab] OR appendiceal mucinous neoplasm*[Tiab] OR colorectal cancer*[Tiab] OR colon cancer*[Tiab] OR colorectal carcinoma*[Tiab] OR colon adenocarcinoma*[Tiab] OR colorectal adenocarcinoma*[Tiab] OR adenomatous polypos*[Tiab] OR gardner syndrome*[Tiab] OR gardner's syndrome*[Tiab] OR peutz jeghers syndrome*[Tiab] OR nonpolypos*[Tiab] OR sigmoid neoplasm*[Tiab] OR sigmoid cancer*[Tiab] OR sigmoid carcinoma*[Tiab] OR sigmoid adenocarcinoma[Tiab] OR recto-sigmoid neoplasm*[Tiab] OR recto-sigmoid cancer*[Tiab] OR recto-sigmoid carcinoma[Tiab] OR rectosigmoid neoplasm*[Tiab] OR rectosigmoid cancer*[Tiab] OR rectosigmoid carcinoma*[Tiab] OR rectal cancer*[Tiab] OR rectum cancer*[Tiab] OR rectal carcinoma*[Tiab] OR rectum carcinoma*[Tiab] OR anorectal cancer*[Tiab] OR anorectal carcinoma*[Tiab] OR anal neoplasm*[Tiab] OR anal cancer*[Tiab] OR anal carcinoma*[Tiab] OR anus neoplasm*[Tiab] OR anus cancer*[Tiab] OR anus carcinoma[Tiab] OR hepatic neoplasm*[Tiab] OR hepatic cancer*[Tiab] OR hepatic carcinoma*[Tiab] OR hepatoid adenocarcinoma*[Tiab] OR hepatocellular carcinoma*[Tiab] OR liver neoplasm*[Tiab] OR liver cancer*[Tiab] OR liver carcinoma*[Tiab] OR liver adenoma*[Tiab] OR hepatic adenoma*[Tiab] OR hepatocellular adenoma*[Tiab] OR pancreas adenoma[Tiab] OR pancreatic adenoma*[Tiab] OR insulinoma*[Tiab] OR islet cell adenoma*[Tiab] OR islet cell tum*[Tiab] OR islet cell carcinoma*[Tiab] OR pancreas neoplasm*[Tiab] OR pancreas cancer*[Tiab] OR pancreatic neoplasm*[Tiab] OR pancreatic cancer*[Tiab] OR pancreatic tum*[Tiab] OR pancreatic neuroendocrine tum*[Tiab] OR pancreatic adenocarcinoma*[Tiab] OR pancreatic ductal adenocarcinoma*[Tiab] OR gastrinoma*[Tiab] OR somatostatinoma*[Tiab] OR vipoma*[Tiab] OR ductal neoplasm*[Tiab] OR ductal cancer*[Tiab] OR ductal carcinoma*[Tiab] OR ductal adenocarcinoma*[Tiab] OR peritoneal neoplasm*[Tiab] OR peritoneal cancer*[Tiab] OR peritoneal carcinomatos*[Tiab] OR peritoneal metastas*[Tiab] OR barrett*[Tiab] OR barrett's[Tiab] OR crest syndrome*[Tiab] OR esophageal achalasia[Tiab] OR esophageal spasm*[Tiab] OR reflux*[Tiab] OR plummer vinson syndrome[Tiab] OR esophageal diverticulosis[Tiab] OR varices[Tiab] OR esophageal cyst*[Tiab] OR esophageal perforation*[Tiab] OR mallor*[Tiab] OR mallory weiss*[Tiab] OR esophageal stenos*[Tiab] OR oesophagitis[Tiab] OR appendicitis[Tiab] OR cholera*[Tiab] OR enterocolitis[Tiab] OR typhlitis[Tiab] OR proctitis[Tiab] OR proctocolitis[Tiab] OR diverticu*[Tiab] OR meckel's[Tiab] OR dysenter*[Tiab] OR enteritis*[Tiab] OR duodenitis*[Tiab] OR ileitis[Tiab] OR pouchitis*[Tiab] OR gastritis*[Tiab] OR gastroenteritis*[Tiab] OR inflammatory bowel[Tiab] OR colitis[Tiab] OR crohn's*[Tiab] OR crohn*[Tiab] OR mucositis[Tiab] OR coeliac*[Tiab] OR gastrointestinal hemorrhage*[Tiab] OR gastrointestinal bleeding*[Tiab] OR hematemes*[Tiab] OR melena*[Tiab] OR peptic ulcer*[Tiab] OR chilaiditi syndrome[Tiab] OR pseudo obstruction*[Tiab] OR irritable bowel*[Tiab] OR neurogenic bowel*[Tiab] OR megacolon*[Tiab] OR Hirschsprung*[Tiab] OR duodenal obstruction*[Tiab] OR superior mesenteric artery syndrome*[Tiab] OR duodenal ulcer*[Tiab] OR gastric ulcer*[Tiab] OR stomach ulcer*[Tiab] OR ulcer perforation*[Tiab] OR zollinger*[Tiab] OR enteropath*[Tiab] OR anisakiasis[Tiab] OR balantidiasis[Tiab] OR blastocystis*[Tiab] OR cryptosporidios*[Tiab] OR dientamoebiasis[Tiab] OR giardias*[Tiab] OR afferent loop obstruction[Tiab] OR afferent loop syndrome*[Tiab] OR fecal impaction*[Tiab] OR fecal obstruction[Tiab] OR volvulus[Tiab] OR intussusception*[Tiab] OR ileus*[Tiab] OR intestinal perforation*[Tiab] OR blind loop syndrome*[Tiab] OR celiac disease*[Tiab] OR collagenous sprue[Tiab] OR lactose intolerance[Tiab] OR short bowel syndrome*[Tiab] OR steatorrhea*[Tiab] OR whipple disease[Tiab] OR whipple's disease[Tiab] OR mesenteric ischemia*[Tiab] OR mesenteric vascular occlusion*[Tiab] OR mesenteric artery occlusion*[Tiab] OR mesenteric artery embolism*[Tiab] OR pneumatosis cystoides intestinalis[Tiab] OR anal fissure*[Tiab] OR pruritus ani[Tiab] OR fecal incontinence*[Tiab] OR hemorrhoid*[Tiab] OR rectal prolapse*[Tiab] OR rectocele*[Tiab] OR rumination*[Tiab] OR achlorhydria*[Tiab] OR bile reflux*[Tiab] OR gastric ectasia*[Tiab] OR gastric dilatation*[Tiab] OR gastric outlet obstruction*[Tiab] OR pyloric stenos*[Tiab] OR gastroparesis[Tiab] OR postgastrectomy syndrome*[Tiab] OR dumping syndrome*[Tiab] OR stomach rupture*[Tiab] OR gastric rupture*[Tiab] OR gastrointestinal tuberculosis[Tiab] OR visceral prolapse[Tiab] OR alpha 1-antitrypsin deficienc*[Tiab] OR budd chiari*[Tiab] OR drug induced liver injur*[Tiab] OR chemical induced liver injur*[Tiab] OR chemically induced liver injur*[Tiab] OR hepatic necros*[Tiab] OR alagille syndrome*[Tiab] OR liver cirrhos*[Tiab] OR fatty liver*[Tiab] OR reye syndrome[Tiab] OR focal nodular hyperplasia*[Tiab] OR hepatic infarction*[Tiab] OR hepatic insufficiency[Tiab] OR liver insufficiency[Tiab] OR liver failure*[Tiab] OR hepatic encephalopath*[Tiab] OR hepatic veno-occlusive disease*[Tiab] OR hepatit*[Tiab] OR rift valley fever[Tiab] OR hepatolenticular degeneration[Tiab] OR hepatomegal*[Tiab] OR hepatopulmonary syndrome*[Tiab] OR hepatorenal syndrome*[Tiab] OR portal hypertension*[Tiab] OR liver abscess*[Tiab] OR hepatic abscess*[Tiab] OR liver cirrhos*[Tiab] OR hepatic cirrhos*[Tiab] OR peliosis hepatis[Tiab] OR hepatic porphyrias[Tiab] OR hepatic tuberculosis[Tiab] OR zellweger syndrome[Tiab] OR congenital hyperinsulinism[Tiab] OR nesidioblastos*[Tiab] OR cystic fibros*[Tiab] OR pancreatic insufficienc*[Tiab] OR shwachman diamond syndrome[Tiab] OR pancreatic cyst*[Tiab] OR pancreatic pseudocyst*[Tiab] OR pancreatic fistula*[Tiab] OR exocrine pancreatic insufficiency[Tiab] OR pancreatic necros*[Tiab] OR pancreatitis*[Tiab] OR chylous ascites[Tiab] OR hemoperitoneum*[Tiab] OR mesenteric lymphadenitis[Tiab] OR peritoneal panniculitis[Tiab] OR peritoneal fibrosis[Tiab] OR peritoneal neoplasm*[Tiab] OR mesenteric cyst*[Tiab] OR peritonitis*[Tiab] OR subphrenic abscess*[Tiab] OR sepsis[Tiab] OR pneumoperitoneum*[Tiab])

***Embase***

digestive system/ or digestive system disease/ or gastrointestinal symptom/ or gastrointestinal tract/ or sepsis/ or peritonitis/ or biliary peritonitis/ or bacterial peritonitis/ or (digestive system OR digestive disease* OR gastrointestinal OR biliary atresia OR biliary fistura OR cholangitis OR choledochal cyst* OR caroli disease OR cholestas* OR mirizzi OR alagille syndrome* OR biliary dyskinesia OR sphincter of oddi dysfunction OR choledocholithias* OR pancreaticobiliary maljunction* OR anorectal malformation OR imperforate anus OR diaphragmatic eventration* OR esophageal atresia* OR intestinal atresia* OR duodenal atresia* OR median arcuate ligament syndrome* OR esophageal fistula* OR tracheoesophageal fistula* OR gastric fistula* OR intestinal fistula* OR rectal fistula* OR anal fistula* OR perianal fistula* OR rectovaginal fistula* OR gastrointestinal neoplasm* OR gastrointestinal cancer* OR gastrointestinal carcinoma* OR gastrointestinal stromal tum* OR GIST* OR digestive system neoplasm* OR intestinal neoplasm* OR intestinal cancer* OR intestinal carcinoma* OR bile duct neoplasm* OR bile duct cancer* OR bile duct carcinoma* OR biliary tract neoplasm* OR biliary tract cancer* OR biliary tract carcinoma* OR cholangiocellular carcinoma* OR cholangiocarcinoma* OR gallbladder neoplasm* OR gallbladder cancer* OR gallbladder carcinoma* OR gallbladder adenocarcinoma* OR esophageal neoplasm* OR esophageal cancer* OR esophageal carcinoma* OR esophageal squamous cell carcinoma* OR esophageal adenocarcinoma* OR gastric neoplasm* OR gastric cancer* OR carcinoma ventriculi OR gastric carcinoma* OR gastric adenocarcinoma* OR gastric squamous cell carcinoma* OR stomach neoplasm* OR stomach cancer* OR stomach carcinoma* OR duodenal cancer* OR duodenal carcinoma* OR duodenal adenocarcinoma* OR ileal neoplasm* OR ileum carcinoma* OR jejunal neoplasm* OR jejunal carcinoma OR jejunum neoplasm* OR jejunum carcinoma OR cecal neoplasm* OR cecal cancer* OR cecal carcinoma* OR cecal adenocarcinoma* OR appendiceal mucinous neoplasm* OR colorectal cancer* OR colon cancer* OR colorectal carcinoma* OR colon adenocarcinoma* OR colorectal adenocarcinoma* OR adenomatous polypos* OR gardner syndrome* OR gardner's syndrome* OR peutz jeghers syndrome* OR nonpolypos* OR sigmoid neoplasm* OR sigmoid cancer* OR sigmoid carcinoma* OR sigmoid adenocarcinoma OR recto-sigmoid neoplasm* OR recto-sigmoid cancer* OR recto-sigmoid carcinoma OR rectosigmoid neoplasm* OR rectosigmoid cancer* OR rectosigmoid carcinoma* OR rectal cancer* OR rectum cancer* OR rectal carcinoma* OR rectum carcinoma* OR anorectal cancer* OR anorectal carcinoma* OR anal neoplasm* OR anal cancer* OR anal carcinoma* OR anus neoplasm* OR anus cancer* OR anus carcinoma OR hepatic neoplasm* OR hepatic cancer* OR hepatic carcinoma* OR hepatoid adenocarcinoma* OR hepatocellular carcinoma* OR liver neoplasm* OR liver cancer* OR liver carcinoma* OR liver adenoma* OR hepatic adenoma* OR hepatocellular adenoma* OR pancreas adenoma OR pancreatic adenoma* OR insulinoma* OR islet cell adenoma* OR islet cell tum* OR islet cell carcinoma* OR pancreas neoplasm* OR pancreas cancer* OR pancreatic neoplasm* OR pancreatic cancer* OR pancreatic tum* OR pancreatic neuroendocrine tum* OR pancreatic adenocarcinoma* OR pancreatic ductal adenocarcinoma* OR gastrinoma* OR somatostatinoma* OR vipoma* OR ductal neoplasm* OR ductal cancer* OR ductal carcinoma* OR ductal adenocarcinoma* OR peritoneal neoplasm* OR peritoneal cancer* OR peritoneal carcinomatos* OR peritoneal metastas* OR barrett* OR barrett's OR crest syndrome* OR esophageal achalasia OR esophageal spasm* OR reflux* OR plummer vinson syndrome OR esophageal diverticulosis OR varices OR esophageal cyst* OR esophageal perforation* OR mallor* OR mallory weiss* OR esophageal stenos* OR oesophagitis OR appendicitis OR cholera* OR enterocolitis OR typhlitis OR proctitis OR proctocolitis OR diverticu* OR meckel's OR dysenter* OR enteritis* OR duodenitis* OR ileitis OR pouchitis* OR gastritis* OR gastroenteritis* OR inflammatory bowel OR colitis OR crohn's* OR crohn* OR mucositis OR coeliac* OR gastrointestinal hemorrhage* OR gastrointestinal bleeding* OR hematemes* OR melena* OR peptic ulcer* OR chilaiditi syndrome OR pseudo obstruction* OR irritable bowel* OR neurogenic bowel* OR megacolon* OR Hirschsprung* OR duodenal obstruction* OR superior mesenteric artery syndrome* OR duodenal ulcer* OR gastric ulcer* OR stomach ulcer* OR ulcer perforation* OR zollinger* OR enteropath* OR anisakiasis OR balantidiasis OR blastocystis* OR cryptosporidios* OR dientamoebiasis OR giardias* OR afferent loop obstruction OR afferent loop syndrome* OR fecal impaction* OR fecal obstruction OR volvulus OR intussusception* OR ileus* OR intestinal perforation* OR blind loop syndrome* OR celiac disease* OR collagenous sprue OR lactose intolerance OR short bowel syndrome* OR steatorrhea* OR whipple disease OR whipple's disease OR mesenteric ischemia* OR mesenteric vascular occlusion* OR mesenteric artery occlusion* OR mesenteric artery embolism* OR pneumatosis cystoides intestinalis OR anal fissure* OR pruritus ani OR fecal incontinence* OR hemorrhoid* OR rectal prolapse* OR rectocele* OR rumination* OR achlorhydria* OR bile reflux* OR gastric ectasia* OR gastric dilatation* OR gastric outlet obstruction* OR pyloric stenos* OR gastroparesis OR postgastrectomy syndrome* OR dumping syndrome* OR stomach rupture* OR gastric rupture* OR gastrointestinal tuberculosis OR visceral prolapse OR alpha 1-antitrypsin deficienc* OR budd chiari* OR drug induced liver injur* OR chemical induced liver injur* OR chemically induced liver injur* OR hepatic necros* OR alagille syndrome* OR liver cirrhos* OR fatty liver* OR reye syndrome OR focal nodular hyperplasia* OR hepatic infarction* OR hepatic insufficiency OR liver insufficiency OR liver failure* OR hepatic encephalopath* OR hepatic veno-occlusive disease* OR hepatit* OR rift valley fever OR hepatolenticular degeneration OR hepatomegal* OR hepatopulmonary syndrome* OR hepatorenal syndrome* OR portal hypertension* OR liver abscess* OR hepatic abscess* OR liver cirrhos* OR hepatic cirrhos* OR peliosis hepatis OR hepatic porphyrias OR hepatic tuberculosis OR zellweger syndrome OR congenital hyperinsulinism OR nesidioblastos* OR cystic fibros* OR pancreatic insufficienc* OR shwachman diamond syndrome OR pancreatic cyst* OR pancreatic pseudocyst* OR pancreatic fistula* OR exocrine pancreatic insufficiency OR pancreatic necros* OR pancreatitis* OR chylous ascites OR hemoperitoneum* OR mesenteric lymphadenitis OR peritoneal panniculitis OR peritoneal fibrosis OR peritoneal neoplasm* OR mesenteric cyst* OR peritonitis* OR subphrenic abscess* OR sepsis OR pneumoperitoneum*).ti,ab.
